# Cut-off Value for Thyroglobulin Washout Concentration in the Detection of Cervical Lymph Node Metastases in Patients after Thyroidectomy Due to Differentiated Thyroid Cancer

**DOI:** 10.3390/biomedicines11092433

**Published:** 2023-08-31

**Authors:** Paweł Mikosiński, Emilia Wołowiec-Korecka, Lech Pomorski, Agnieszka Mikosińska, Krzysztof Kaczka, Sławomir Mikosiński

**Affiliations:** 1 Department of General and Oncological Surgery, University Hospital and Education Centre, Medical University of Lodz, ul. Pomorska 251, 92-231 Lodz, Poland; p.mik@op.pl (P.M.); krzysztof.kaczka@umed.lodz.pl (K.K.); 2 Institute of Materials Science and Engineering, Faculty of Mechanical Engineering, Lodz University of Technology, B. Stefanowskiego 1/15, 90-537 Lodz, Poland; emilia.wolowiec-korecka@p.lodz.pl; 3 Department of General and Oncological Surgery, Maria Sklodowska-Curie Memorial District Hospital, ul. Parzeczewska 35, 95-100 Zgierz, Poland; pomorskil@op.pl; 4 Faculty of Medicine, Medical University of Lodz, al. T. Kosciuszki 4, 90-419 Lodz, Poland; agnieszka.mikosinska@stud.umed.lodz.pl; 5 Department of Nuclear Medicine and Oncological Endocrinology, Maria Sklodowska-Curie Memorial District Hospital, ul. Parzeczewska 35, 95-100 Zgierz, Poland

**Keywords:** thyroglobulin washout concentration cut-off value, differentiated thyroid cancer (DTC), cervical lymph node metastases, fine needle aspiration thyroglobulin (FNA-Tg), fine needle aspiration biopsy cytology (FNAB-C)

## Abstract

The aim of this study was to establish the cut-off value for the thyroglobulin (Tg) concentration in washout fluid from fine needle aspiration biopsy (FNA-Tg) in the detection of cervical lymph node metastases of differentiated thyroid cancer (DTC). We evaluated the validity and clinical utility of fine needle aspiration biopsy cytology (FNAB-C), FNA-Tg, and the combined method in detecting DTC recurrences. The study included 82 patients after the total thyroidectomy and elective and, in some cases, also selective cervical lymphadenectomy. The majority of patients also underwent subsequent ^131^I ablative therapy. The patients presented with 1–6 enlarged and/or ultrasonographically suspicious cervical lymph nodes. One to four aspirates of each lymph node were taken, with a total of 297 samples. An FNA-Tg of 4.34 ng/mL was established as the cut-off value for detecting cervical lymph node DTC metastases for the IRMA Brahms DYNO test, Tg-S. FNAB-C is highly specific (91–99%) but not sensitive enough (53–69%) to be used as a standalone method in the detection of cervical lymph node metastases. FNA-Tg is more sensitive (91%), but caution should be taken when selecting patients for surgery with an FNA-Tg higher than the established cut-off value but lower than the serum Tg concentration. To select patients for lymphadenectomy, we recommend using the combined method (FNAB-C and FNA-Tg) with a sensitivity of 96% and specificity of up to 97%. More than one sample should be taken with each fine needle aspiration biopsy (FNAB) to obtain a representative set of samples.

## 1. Introduction

Thyroid cancer is the most often observed endocrine malignancy. It accounts for 96% of all endocrine organ carcinomas [1]. Derived from follicular epithelial cells, differentiated thyroid cancer (DTC), which constitutes over 90% of all thyroid malignancies, can be subdivided into papillary thyroid carcinoma (PTC; 85–90%), follicular thyroid carcinoma (FTC; 5–10%), and oncocytic carcinoma of the thyroid (OCA; 2–5%)—the rare type, formerly called Hürthle cell thyroid carcinoma [2,3,4]. Poorly differentiated thyroid carcinoma (PDTC), also originating from follicular epithelial cells, is an intermediate entity between DTC and anaplastic thyroid carcinoma (ATC), which accounts for 3–5% of all thyroid carcinomas [5]. In the 2022 WHO classification, PDTC was categorized as a high-grade follicular cell-derived carcinoma [3,4]. DTC is approximately three times more common in females than males, occurs very often at a young age, and has an excellent prognosis, with a 5-year relative survival rate of 98.3% [2]. This favorable outcome is mainly due to early diagnosis, good response to surgical and postoperative radioiodine (^131^I) treatment, and effective methods of monitoring and detecting recurrent/persistent disease. While the prognosis is generally good, up to 20% of patients present with local or regional recurrences during follow-up after the initial surgery [6,7,8]. Since cervical lymph nodes are the most common site of metastases, it is recommended to perform an ultrasound neck examination every 6–12 months for the first 5 years after thyroidectomy, with possibly longer intervals later on [9]. Ultrasonography is highly sensitive in the detection of cervical lymph node metastases but has low specificity due to frequently occurring benign lymphadenopathies [10]. Suspicious ultrasonographic features of malignant lymph nodes include cystic degeneration (litho-cystic appearance), punctate calcium flecks (microcalcifications), increased cortical echogenicity (hyperechoic content), absence of hilum (loss of the hilar line), enlarged node size (>5 mm in height), a round shape with a >0.5 height/width ratio in the transverse view (Solbiati index—maximal long/short axis <2), peripheral or diffuse hypervascularity, and reduced node elasticity—in the case of multiple abnormal nodes. Such lymph nodes are those with a higher probability of metastases, and fine needle aspiration biopsy (FNAB) with cytology (FNAB-C) should be performed [9,11,12,13,14,15,16].

Thyroglobulin (Tg) is a glycoprotein produced by the follicular cells of the thyroid gland and also by all types of DTC cells. Measurement of the Tg serum concentration can detect recurrent/persistent disease in DTC patients who underwent total thyroidectomy and cervical lymphadenectomy with or without subsequent ^131^I treatment. Tg concentration in the washout fluid from lymph node fine needle aspiration biopsy (fine needle aspiration thyroglobulin, FNA-Tg) is used to detect cervical lymph node metastases [17]. Circulating serum anti-Tg antibodies (aTg) may interfere with the serum Tg and FNA-Tg measurements, resulting in lower Tg concentrations; however, the clinical performance of FNA-Tg appears not to be substantially affected by aTg; thus, FNA-Tg measurement remains highly reliable in the detection of DTC cervical lymph node metastases, even in patients with positive aTg [18,19].

The measurement of FNA-Tg is recommended in both the American and the European Thyroid Association Guidelines and has been reported to complement FNAB-C in the detection of DTC recurrences [20,21,22,23,24,25]. In the 2015 ATA guidelines, an FNA-Tg > 10 ng/mL is considered the threshold of high suspicion of malignancy [24]. However, there is a need for ongoing research due to the lack of explicit international consensus regarding FNA-Tg sample collection protocols and the absence of a defined method-specific laboratory cut-off value for FNA-Tg.

The aim of this study was to evaluate FNA-Tg and establish a cut-off value for cervical lymph node metastases in DTC patients after a total thyroidectomy with elective and, in some cases, also selective cervical lymphadenectomy. We also evaluated the validity and clinical utility of FNAB-C, FNA-Tg, and their combination (combined method) in detecting recurrence.

## 2. Materials and Methods

This is a retrospective study. A total of 82 patients (54 females and 28 males), aged 17–83 years (with a mean age of 48.1 years) were studied; 71 of them had PTC, 8 FTC, 1 OCA, 1 PDTC, and 1 mixed type carcinoma—PTC subtype solid/FTC. All patients had undergone total thyroidectomy with routine prophylactic (elective) central cervical (level VI) lymphadenectomy and, in some cases, also selective neck dissection (levels II–IV). The majority of patients also underwent subsequent ^131^I ablative therapy. In an ultrasound examination, they presented with 1–6 enlarged and/or suspicious cervical lymph nodes. Ultrasound-guided (linear probe, 7.27/8 MHz, Aloka Prosound Alpha 6, Japan or linear probe 7 to 14 MHz Aplio 500 Toshiba Medical Systems, Otawara, Japan) standard FNAB was performed in all patients, taking 1–4 aspirates of each lymph node and obtaining a total of 297 samples (at least two aspirates/samples taken if solitary lymph node tested). The material collected in the needle (0.5 mm thick, 2.5 or 4 cm long; 25 gauge) was expelled onto a slide for cytologic analysis (FNAB-C). Every used FNAB needle was washed out with 125 µL of 0.9% saline, and the Tg concentration (FNA-Tg) and Tg recovery were measured using the same Tg kit as used for serum measurements, i.e., the immunoradiometric assay (IRMA) Brahms DYNO test Tg-S; BRAHMS Diagnostica, GmbH, Berlin, Germany.

The study patients were mostly under endogenous TSH stimulation conditions (TSH 0.01–99.95 µIU/mL; mean 60.80 µIU/mL; median 60.11 µIU/mL) achieved within at least four weeks after withdrawal of *Levothyroxine* (LT_4_) therapy. TSH stimulation conditions are not necessary for performing FNAB with FNA-Tg measurement; however, the study patients were admitted for routine clinical check-ups with a ^131^I whole body scan (WBS) scheduled. The latter procedure is reliable only under TSH stimulation conditions.

All 82 patients were either treated with surgery again or underwent 5-year active surveillance after primary surgery. Patients were selected for reoperation based on their malignant FNAB-C and/or elevated FNA-Tg status (>5 ng/mL). In the absence of specific indications (standards) regarding the applicability of these criteria and the lack of a defined method-specific laboratory cut-off value for FNA-Tg, patient eligibility decisions relied on the clinical experience of the medical team (researcher performing FNAB and surgeon), supported by worldwide literature. Patients who did not fulfill these criteria underwent 5-year active surveillance after primary surgery.

Thirty-six patients with malignant FNAB-C and/or high (>5 ng/mL) FNA-Tg (in at least one sample) were treated with surgery again. Subsequent pathology exams were performed on excised lymph nodes. One hundred thirty-six samples of FNAB-C and FNA-Tg (collected from these patients prior to surgery) were matched with corresponding pathology results after reoperation.

Forty-five patients with benign or non-diagnostic FNAB-C and low (<5 ng/mL) FNA-Tg (in all samples from each patient) were not reoperated. No corresponding pathology reports were available for 155 samples of FNAB-C and FNA-Tg collected from these patients. They underwent 5-year active surveillance and showed no evidence of recurrence.

One patient had two suspicious lymph nodes, and only one of them was excised during surgery. Four samples with malignancy in FNAB-C and high FNA-Tg (obtained prior to surgery from the excised lymph node) had one corresponding pathology result. The second suspicious lymph node was not excised. Two samples with benign FNAB-C but with elevated FNA-Tg (from the non-excised lymph node) had no corresponding pathology result available. Later, the patient underwent 5-year active surveillance and showed no evidence of recurrence.

Generally, lymph nodes are excised as en bloc resection. Sometimes, node “plucking” or “berry picking”, i.e., removing only the suspicious lymph nodes, may be performed, especially in limited nodal involvement. For the purpose of this approach, we designed, individually for every patient, a special topographic map of the neck with all suspicious lymph nodes marked in red. The map was placed over the patient’s head during the surgery. Despite all this effort, in the case mentioned above, the marked node could have been simply skipped or not found.

A final evaluation was performed on the samples from patients treated with surgery (i.e., 136 + 4 samples with a corresponding pathology result) and samples from patients who completed the 5-year active surveillance with no evidence of recurrence (155 + 2).

Statistical analysis was performed using Statistica software (version 13.0) by StatSoft. A Mann–Whitney U test was used to compare FNA-Tg median values between patients with a malignant pathology result and patients with either a benign pathology result or with no evidence of recurrence after 5-year active surveillance. The FNA-Tg cut-off value for detecting cervical lymph node metastases of DTC was estimated using the Receiver Operating Characteristic (ROC) curve, classification trees analysis, and neural networks. The results of FNAB-C and FNA-Tg were compared to corresponding pathology reports in patients who had undergone surgery or to the final disease status (no evidence of recurrence) in patients who underwent 5-year active surveillance. Diagnostic measures, including sensitivity, specificity, positive predictive value (PPV), negative predictive value (NPV), prevalence (PV), and accuracy (ACC), were calculated separately for cytologic analysis (FNAB-C), FNA-Tg, and the combined method.

## 3. Results

### 3.1. Distribution of Samples

One hundred thirty-six samples (from 36 patients) with malignant FNAB-C and/or high FNA-Tg (>5 ng/mL; range, 0.13–922.80 ng/mL) had corresponding pathology results from excised lymph nodes.

One hundred fifty-five samples (from 45 patients) with benign or non-diagnostic FNAB-C and low FNA-Tg (<5 ng/mL; range, 0.02–4.30 ng/mL) had no corresponding pathology results available. The patients from whom these samples were obtained underwent 5 years of active surveillance and showed no evidence of recurrence.

Four samples (from one patient with two suspicious lymph nodes and only one excised) with malignancy in the FNAB-C and high FNA-Tg (58.84 ng/mL; 146.64 ng/mL; 361.51 ng/mL; 242.23 ng/mL) had a corresponding pathology result, confirming the presence of metastasis in the operated lymph node. Two samples, originating from the non-excised lymph node, with benign FNAB-C but with elevated FNA-Tg (9.16 ng/mL; 11.85 ng/mL), had no corresponding pathology result available. Later, the patient underwent 5 years of active surveillance and showed no evidence of recurrence.

**Figure 1:**
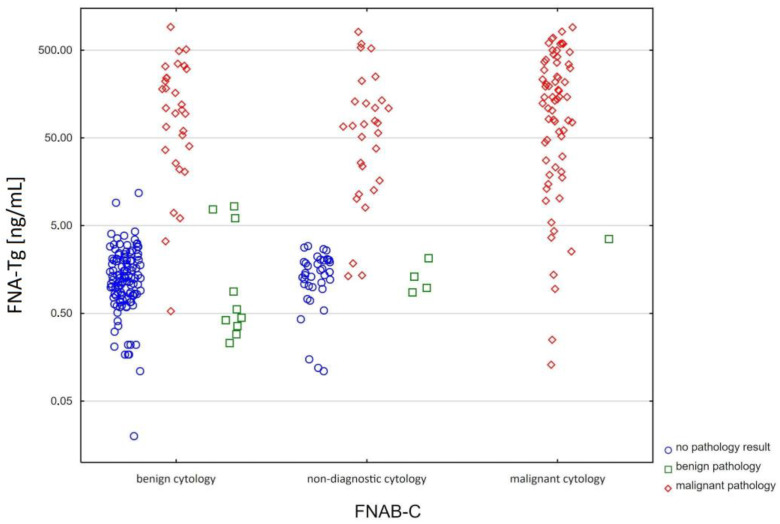
Distribution of samples.

Figure 1 shows the distribution of the samples, depending on the cytology result (FNAB-C), FNA-Tg, and corresponding pathology result.

The blue circles represent samples with no pathology report available. The green squares represent samples with a benign pathology result, and the red rhombuses represent samples with a malignant pathology result.

Out of the 67 samples with malignant FNAB-C, sixty-six were confirmed as originating from malignant lymph nodes in the postoperative pathology exam, and only one from a benign lymph node.

Out of all of the two hundred ninety-seven samples, five, which were benign (2/5) or non-diagnostic (3/5) in the FNAB-C and with low FNA-Tg (<5 ng/mL), were verified as originating from malignant lymph nodes in the postoperative pathology exam. The patients were eligible for surgery because they had an additional cytologically malignant adenopathy and/or high FNA-Tg.

Out of all of the two hundred ninety-seven samples, eleven, which were benign (7/11) or non-diagnostic (4/11) in the FNAB-C and with low FNA-Tg (<5 ng/mL), were confirmed as originating from benign lymph nodes in the postoperative pathology exam. The patients were eligible for surgery because they had an additional cytologically malignant adenopathy and/or high FNA-Tg.

Seventy out of all of the two hundred ninety-seven samples were non-diagnostic in the FNAB-C—23.6%. Twenty-nine out of seventy (41.4%) non-diagnostic samples in the FNAB-C turned out to originate from malignant lymph nodes in the postoperative pathology exam.

### 3.2. FNA-Tg Evaluation

In this study, the patients’ serum Tg concentration was within the 0.04–711.5 ng/mL range (mean 27.02 ng/mL; median 3.40 ng/mL), and the Tg recovery range was 92–100%. Serum aTg were within the 2–150 IU/mL range (mean 29.26 IU/mL; median 25 IU/mL; laboratory reference range 0–40 IU/mL).

**Figure 2:**
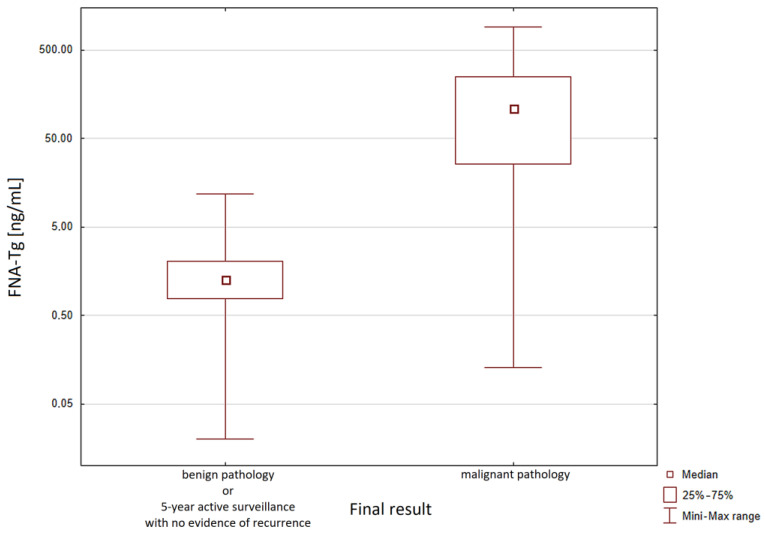
FNA-Tg ranges and medians of samples corresponding to malignant pathology group and to group with benign pathology or no evidence of recurrence after 5-year active surveillance.

A final evaluation of the FNA-Tg was performed on samples from patients treated with surgery (i.e., samples with a corresponding pathology result) and samples from patients who completed the 5 years of active surveillance with no evidence of recurrence. The median FNA-Tg in patients with a malignant pathology result was higher than the median FNA-Tg in patients with either a benign pathology result or no evidence of recurrence after the 5 years of active surveillance (Figure 2).

The FNA-Tg range in the malignant pathology group was 0.13–922.80 ng/mL, with a median value of 108.66 ng/mL. In the group with benign pathology or no evidence of recurrence after the 5 years of active surveillance, the FNA-Tg range was 0.02–11.85 ng/mL, with a median value of 1.28 ng/mL. Statistical analysis (Mann–Whitney U test) revealed a statistically significant difference between the FNA-Tg median values in these two groups (*p* < 0.0001).

### 3.3. FNA-Tg Cut-Off for Detecting Cervical Lymph Node Metastases of DTC

**Figure 3:**
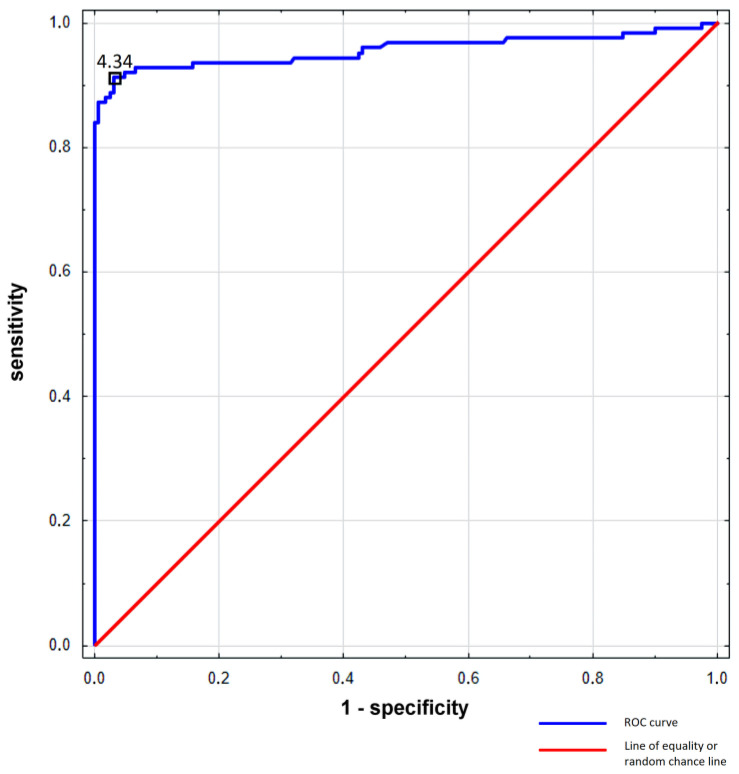
The ROC curve of FNA-Tg.

The FNA-Tg cut-off for detecting cervical lymph node metastases of DTC was estimated using the Receiver Operating Characteristic (ROC) curve, classification trees analysis, and neural networks. The respective results were 4.34 ng/mL, 4.96 ng/mL, and 5.0 ng/mL. Of all three statistical methods used, the ROC curve was considered the most relevant. For further analysis, we established the FNA-Tg cut-off value of 4.34 ng/mL. The ROC curve is shown in Figure 3.

### 3.4. Diagnostic Measures for FNAB-C, FNA-Tg and the Combined Method

The sensitivity, specificity, positive predictive value (PPV), negative predictive value (NPV), prevalence (PV), and accuracy (ACC) were estimated separately for cytologic analysis (FNAB-C), FNA-Tg, and the combined method in two groups of patients:

1. All 82 patients (297 samples)—treated with surgery or with active surveillance;
2. 36 patients + 1* (136 samples + 4*)—only patients treated with surgery and with a postoperative pathology report.

* the patient with two suspicious lymph nodes and only one excised (four samples)

Malignant FNAB-C was described as a positive cytology, and benign or non-diagnostic FNAB-C as negative.

For the FNA-Tg evaluation, all samples with an FNA-Tg higher than 4.34 ng/mL were considered positive and below as negative.

The results of the FNAB-C and FNA-Tg were compared to corresponding pathology reports in patients who had undergone surgery. All patients who underwent 5 years of active surveillance showed no evidence of recurrence. The cases are shown in Table 1.

Five out of all 297 samples were false positive for FNA-Tg. Three of those five had a corresponding postoperative pathology report (Figure 1—the green squares in the first column above the 5 ng/mL line). One patient (two samples) who underwent active surveillance was false positive for FNA-Tg (Figure 1—the blue circles in the first column above the 5 ng/mL line), with FNA-Tg concentrations of 9.16 ng/mL and 11.85 ng/mL. The remaining patients (samples) who underwent active surveillance were truly negative (showed no recurrence) for the FNAB-C and FNA-Tg methods (Figure 1—the remaining blue circles).

In the combined method, malignant FNAB-C and/or FNA-Tg above 4.34 ng/mL were described as positive, whereas negative meant benign or non-diagnostic FNAB-C and FNA-Tg below 4.34 ng/mL. The cases are shown in Table 2.

The sensitivity, specificity, positive predictive value (PPV), negative predictive value (NPV), prevalence (PV), and accuracy (ACC), separately for FNAB-C, FNA-Tg, and for the combined method in two groups of patients (1—all samples; 2—samples from patients treated with surgery with postoperative pathology report), are shown in Table 3.

Two groups of patients were analyzed (1—all samples; 2—samples from patients treated with surgery with postoperative pathology report). Because the resulting FNAB-C sensitivity was low (53%), we reassessed all the measures (the sensitivity, specificity, PPV, NPV, PV, and ACC) for the same groups, excluding all samples with non-diagnostic FNAB-C. The cases and measures are shown, respectively, in Table 4 and Table 5.

When the analysis was limited to only samples with diagnostic FNAB-C (benign or malignant) in the two groups of patients (1—all samples; 2—samples from patients treated with surgery with a postoperative pathology report), the sensitivity of FNAB-C increased to 69%.

## 4. Discussion

The outcomes of our study indicate that despite being highly specific (91–99%), FNAB-C is not sensitive enough (53–69%) to be used as a standalone method for detecting cervical lymph node metastases of DTC. There was only one patient (one sample) out of eighty-two patients (two hundred ninety-seven samples) with a false positive diagnosis in the FNAB-C. The patient underwent subsequent left-side neck lymphadenectomy. The routine postoperative pathology exam showed no evidence of thyroid cancer cells in any of the structures excised during surgery. The pathology results were validated in a serial examination of the excised structures. That the lymph node removed was indeed the proper one was confirmed in an ultrasound examination a few weeks after the surgery. We set out to explain the discrepancy between FNAB-C and the pathology result, which was not observed in our previous study [26].

One possibility involves an incorrect assessment of the cytologic sample. In this case, the cytology result was formulated in Latin. It read “Cellulae carcinomatis papillaris glandulae thyroideae non multae”, which means: “not many papillary thyroid cancer cells” and is a rather uncommon description. Another explanation (very unlikely, but theoretically possible) is that all of the malignant cells were aspirated into the needle during the FNAB. The third reason might be that the malignant cells underwent apoptosis or phagocytosis by macrophages during the time between the FNAB and the surgery.

Since the last explanation appeared as the most probable, we decided to take a closer look at the case. It turned out that this patient had been treated with ^131^I 80 days before the FNAB, receiving activity of 144 mCi (5328 MBq) due to an elevated serum Tg concentration (16.56 ng/mL). It was her fourth and final treatment. Overall, she received a total of 511 mCi (18.907 GBq) ^131^I activity. Forty-seven days after the FNAB, she underwent a lymphadenectomy, and it was her fifth and final surgery. During the period between the ^131^I treatment and the surgery, the irradiated cancer cells could have already been eliminated [27]. The patient has remained disease-free (no suspicious lymph nodes in ultrasound exams, negative in ^131^I whole body scans, a Tg serum concentration of <1.0 ng/mL in Brahms DYNO test Tg-S, or undetectable in other laboratory tests) in the 6-year follow-up until now.

The one patient who had undergone surgery with the removal of only one of two suspicious lymph nodes was placed under 5 years of active surveillance and showed no evidence of recurrence. The two samples obtained prior to surgery from the non-excised lymph node demonstrated benign FNAB-C but elevated FNA-Tg (9.16 ng/mL; 11.85 ng/mL) and were therefore categorized as false positive in the FNA-Tg and the combined method. We found that preoperative FNA-Tg levels were much higher in the excised lymph node (four samples: 58.84 ng/mL; 146.64 ng/mL; 361.51 ng/mL; 242.23 ng/mL) compared to the non-excised lymph node (two samples: 9.16 ng/mL; 11.85 ng/mL). Although the Tg protein particle alone (not as a part of the cancer cell) can be drained through the lymphatic system, it does not have to explain the false positive result in this case [26,28]. At this point, the serum Tg concentration was 24.9 ng/mL—more than twice the level of FNA-Tg in the non-excised lymph node. It is possible that the lymph node was indeed non-metastatic, and the elevated FNA-Tg could have resulted from the needle being contaminated with serum on its passage through the tissues during the FNAB. Therefore, we suggest that caution should be taken when selecting patients for surgery with an FNA-Tg higher than the established cut-off value but lower than the serum Tg concentration.

The remaining patients (samples) who underwent active surveillance had an FNA-Tg below 4.34 ng/mL, showed no evidence of recurrence, and, therefore, were categorized as truly negative in the FNA-Tg method and in the combined method.

There was one patient in whom three samples of one suspicious lymph node were obtained and who was selected for surgery based on one malignant FNAB-C and elevated FNA-Tg (14.86 ng/mL). The remaining two samples were non-diagnostic in the FNAB-C with low FNA-Tg (1.4 ng/mL; 0.53 ng/mL). The patient underwent surgery, and the postoperative pathology result turned out to be positive. Although the physician who performed the ultrasound-guided FNAB was highly experienced (over 700 patients, over 1500 lymph nodes biopsied, and over 3000 samples taken), the two samples mentioned above (non-diagnostic in FNAB-C and with low FNA-Tg) might have been false negative for the simple reason of mistargeting the suspicious lymph node and aspirating material from the surrounding tissues. Since we consider this a highly reasonable explanation, we recommend that more than one sample should be taken with each FNAB to obtain a representative set of samples.

Depending on the study, the sensitivity of FNA-Tg varies due to discrepancies in the established cut-off values and ranges from 81.4% to 100%, while the sensitivity of FNAB-C ranges from 55% to 85% [6,7,18,19,21,26,29,30,31,32,33]. The present study confirms the high sensitivity of FNA-Tg and lower sensitivity of FNAB-C in the detection of DTC cervical lymph node metastases. Our calculated sensitivity of FNA-Tg was 91% and thus fit in the range previously described in various clinical trials. The calculated sensitivity of FNAB-C for all tested samples was 53%, which is below the stated range; however, when the analysis was limited to only samples with diagnostic FNAB-C, the resulting sensitivity (69%) was within the range described. In other studies, the FNAB needle was washed out with a dilutant volume ranging from 195 µL to 3 mL [6,7,16,17,21,22,34,35]. Based on our previous experience [26], in the present study, material collected in the FNAB needle was washed out with a relatively small volume of dilutant (125 µL of 0.9% NaCl), i.e., a volume sufficient for the laboratory test to be performed. The minimal volume of dilutant and resulting higher FNA-Tg concentration may possibly increase the sensitivity of the method. Although 1 mL of normal saline is widely used, we would recommend reducing the volume of the dilutant to the minimum required for laboratory testing.

FNA-Tg was reported to have lower sensitivity and a negative predictive value in the detection of DTC cervical lymph node metastases in a group of patients with positive aTg compared to a group with negative aTg. Even though high-serum aTg could interfere with FNA-Tg, resulting in falsely low results, FNA-Tg remains a highly reliable method [18,19]. In our research, the serum aTg of the study patients were mostly within the laboratory reference range (0–40 IU/mL), with a mean of 29.26 IU/mL, and a median of 25 IU/mL. The Tg recovery range was 92–100%. Therefore, the serum aTg’s influence on the sensitivity of the method is negligible.

The study was conducted in 82 patients with DTC who underwent total thyroidectomy with routine prophylactic (elective) central cervical (level VI) lymphadenectomy and, in some cases, also selective neck dissection (levels II–IV). The group was heterogeneous in regard to the type of differentiated thyroid cancer (seventy-one PTC, eight FTC, one OCA, one PDTC, and one mixed type carcinoma—PTC subtype solid/FTC). However, the number of study patients might be considered a limitation. Furthermore, some studies show that diagnostic measures and cut-off values for FNA-Tg may be affected by the neck region of the tested lymph node and may differ between the central and lateral cervical lymph nodes [34]. The substantial variation in FNAB procedures and FNA-Tg sample preparation protocols contribute significantly to discrepancies between the cut-off values reported in different studies [20,31,35,36,37]. Despite these limitations, our study has valuable clinical implications, proving that FNA-Tg is a valid method for the detection of DTC cervical lymph node metastases. Further large-scale clinical trials should be performed to establish the FNA-Tg cut-off values, specifically for central and lateral cervical lymph nodes; however, our standardized technique, i.e., the type of needle, small volume of dilutant, and Tg kit used for the FNA-Tg measurement, offers a reliable standard operating procedure for future analysis. A consensus regarding standardization of the method should be achieved to adopt a protocol of FNA-Tg sample collection, thereby enabling comparison of the data among studies from different centers.

## 5. Conclusions

In our study, we established the cut-off value for FNA-Tg (IRMA Brahms DYNO test Tg-S) at the level of 4.34 ng/mL with a sensitivity of 91% and a specificity of 80–97% in detecting cervical lymph node metastases of DTC.

FNAB-C is highly specific (91–99%) but not sensitive enough (53–69%) to be used as a standalone method for detecting cervical lymph node metastases of DTC. The results turned out to be non-diagnostic in 23.6%. If FNAB-C is non-diagnostic, FNA-Tg is particularly valuable.

To provide an optimal cure for the largest number of patients with cervical lymph node metastases of DTC, we recommend using the combination of FNAB-C and FNA-Tg in selecting patients for lymphadenectomy. While preserving high specificity (up to 97%), the combined method (FNAB-C and FNA-Tg) provides higher sensitivity (96%) than either of the components used separately.

More than one sample should be taken with each FNAB to obtain a representative set of samples.

## Figures and Tables

**Table 1:** The results of FNAB-C and FNA-Tg evaluation.

	FNAB-C ^1^	FNAB-C ^2^	FNA-Tg ^1^	FNA-Tg ^2^
true positive (TP)	66	66	114	114
false positive (FP)	1	1	5	3
false negative (FN)	59	59	11	11
truly negative (TN)	171	14	167	12
total	297	140	297	140

^1^ FNAB-C—all samples; ^2^ FNAB-C—samples from patients treated with surgery with postoperative pathology report; ^1^ FNA-Tg—all samples; ^2^ FNA-Tg—samples from patients treated with surgery with postoperative pathology report.

**Table 2:** The results of combined method (FNAB-C + FNA-Tg) evaluation.

	Combined Method (FNAB-C + FNA-Tg) ^1^	Combined Method (FNAB-C + FNA-Tg) ^2^
true positive (TP)	120	120
false positive (FP)	6	4
false negative (FN)	5	5
truly negative (TN)	166	11
total	297	140

^1^ Combined method (FNAB-C + FNA-Tg)—all samples; ^2^ Combined method (FNAB-C + FNA-Tg)—samples from patients treated with surgery with postoperative pathology report.

**Table 3:** Calculated diagnostic measures for FNAB-C, FNA-Tg, and combined method.

	FNAB-C ^1^	FNAB-C ^2^	FNA-Tg ^1^	FNA-Tg ^2^	Combined Method (FNAB-C + FNA-Tg) ^1^	Combined Method (FNAB-C + FNA-Tg) ^2^
sensitivity	53%	53%	91%	91%	96%	96%
specificity	99%	93%	97%	80%	97%	73%
PPV	0.99	0.99	0.96	0.97	0.95	0.97
NPV	0.74	0.19	0.94	0.52	0.97	0.69
PV	0.42	0.89	0.42	0.89	0.42	0.89
ACC	0.80	0.57	0.95	0.90	0.96	0.94

^1^ FNAB-C—all samples; ^2^ FNAB-C—samples from patients treated with surgery with postoperative pathology report; ^1^ FNA-Tg—all samples; ^2^ FNA-Tg—samples from patients treated with surgery with postoperative pathology report; ^1^ combined method (FNAB-C + FNA-Tg)—all samples; ^2^ combined method (FNAB-C + FNA-Tg)—samples from patients treated with surgery with postoperative pathology report.

**Table 4:** The results of FNAB-C evaluation, excluding non-diagnostic samples.

	FNAB-C ^1^	FNAB-C ^2^
true positive (TP)	66	66
false positive (FP)	1	1
false negative (FN)	30	30
truly negative (TN)	130	10
total	227	107

^1^ FNAB-C—all samples excluding non-diagnostic; ^2^ FNAB-C—samples from patients treated with surgery with postoperative pathology report, excluding non-diagnostic samples.

**Table 5:** Calculated diagnostic measures for FNAB-C, excluding non-diagnostic samples.

	FNAB-C ^1^	FNAB-C ^2^
sensitivity	69%	69%
specificity	99%	91%
PPV	0.99	0.99
NPV	0.81	0.25
PV	0.42	0.90
ACC	0.86	0.71

^1^ FNAB-C—all samples excluding non-diagnostic; ^2^ FNAB-C—samples from patients treated with surgery with postoperative pathology report, excluding non-diagnostic samples.

## Data Availability

Data are unavailable due to patient privacy.
